# Ferroptosis-related lncRNA signature predicts the prognosis and immune microenvironment of hepatocellular carcinoma

**DOI:** 10.1038/s41598-022-10508-1

**Published:** 2022-04-22

**Authors:** Chongkai Fang, Silin Liu, Kunliang Feng, Chaoyuan Huang, Ying Zhang, Jinan Wang, Hongtong Lin, Junyan Wang, Chong Zhong

**Affiliations:** 1 First Clinical Medical College, Guangzhou University of Chinese Medicine, Guangzhou, 510403 Guangdong China; 2 Lingnan Medical Research Center of Guangzhou University of Chinese Medicine, Guangzhou, 510403 Guangdong China; 3 Department of Hepatobiliary Surgery, First Affiliated Hospital of Guangzhou University of Chinese Medicine, Guangzhou, 510405 Guangdong China; 4 Guangdong Provincial Hospital of Traditional Chinese Medicine, Guangzhou, 510120 Guangdong China; 5 School of Pharmaceutical Sciences, Guangzhou University of Chinese Medicine, Guangzhou, 510006 Guangdong China

**Keywords:** Cancer genomics, Cancer

## Abstract

This study aimed to construct a ferroptosis-related lncRNA signature to probe the prognosis and immune infiltration of HCC patients. The Cancer Genome Atlas (TCGA) database was randomly divided into two parts, with two-thirds training and one-third testing sets. Univariate, multivariate, and least absolute selection operator (LASSO) Cox regression analyses were performed to establish a ferroptosis-related lncRNA signature. The prognostic signature was constructed by 6 ferroptosis-related lncRNAs (PCAT6, MKLN1-AS, POLH-AS1, LINC00942, AL031985.3, LINC00942) shows a promising clinical prediction value in patients with HCC. Patients with high-risk score indicated a poorer prognosis than patients with low-risk score were shown in the training set (*p* < 0.001) and testing set (*p* = 0.024). Principal component analysis (PCA) and nomogram were performed to verify the value of the prognostic signature. The area under curves (AUCs) for 1-, 3-, and 5-year survival rates were 0.784, 0.726, 0.699, respectively. Moreover, TCGA revealed that immune cell subpopulations and related functions, including cytolytic activity, MHC class I, type I and type II IFN response, were significantly different between the two risk groups. Immune checkpoints such as PDCD1, CTLA4, CD44, VTCN1 were also abnormally expressed between the two risk groups. This prognostic signature based on the ferroptosis-related lncRNAs may be promising for the clinical prediction of prognosis and immunotherapeutic responses in patients with HCC.

## Introduction

Hepatocellular carcinoma (HCC) is the sixth most common cancer and the third leading cause of death worldwide^1^. Despite various therapeutic options, including surgery, interventional therapy, targeted therapy, and immunotherapy, which has been performed for HCC, the 5-year overall survival (OS) in HCC patients has remained unsatisfactory because of the high tumor recurrence and metastasis rates^2,3^. Immunotherapy is an emerging treatment attracting the attention of oncologists. In the Phase III study of Imbrave150, Atezolizumab combined with Bevacizumab in advanced HCC reduced the risk of death by 42% and tumor progression by 41%^4^. However, pembrolizumab corresponding to KEYNOTE-240 and nivolumab corresponding to CheckMate-459 did not significantly improve the progression-free survival (PFS) and OS of HCC patients^5,6^. Although immunotherapy has a significant effect on HCC patients, it is crucial to identify effective biomolecular predictors to select immune benefit people. Wang found that immunosuppressants can damage cancer cells more effectively by promoting ferroptosis^7^.

Pathological cell death is implicated in the development of many pathogeneses of cancers. Ferroptosis is a form of iron-dependent cell death characterized by the accumulation of lipid peroxides to lethal levels^8^. After ferroptosis was first identified, ferroptosis was detected in various immune cells, which affects the immune response^7,9^. It is possible to conjecture that ferroptosis and the immune response can interact in some cases. The imbalance of iron metabolism is a risk factor for cancer and can also promote the tumor growth. In late years, ferroptosis has excellent potential for tumorigenesis and cancer therapeutics efficacy^10–12^. A group of scientists first characterized ferroptosis as a novel anti-tumor mechanism, indicating that this process can be performed as a target for cancer immunotherapy^7^. Increasing studies have revealed that sorafenib could induce ferroptosis and improve its resistance in HCC^13,14^, and growing evidence indicates some signal transduction pathways about how ferroptosis is involved in the progression of HCC^15–17^. Actually, the activation of ferroptosis pathways may surpass the resistance of current chemotherapeutics and open up a new frontier for cancer treatment.

Long-noncoding RNAs (lncRNAs) are RNAs that do not code for proteins. The transcription length exceeds 200 nucleotides and lacks a complete open reading frame (ORF). Furthermore, lncRNAs play a vital role in the progression of various cancers^18–20^. H19, HULC, HEIH, linc00152, and MVIH, are highly upregulated lncRNAs and valuable biomarkers in HCC^21^. One recent study revealed that overexpression of LINC00618 promotes cell apoptosis and ferroptosis^22^. The lncRNA P53RRA promotes ferroptosis to suppress cancer progression, further verifying the close relationship between lncRNA and ferroptosis^23^. Another study showed a mechanistic link between GABPB1 and its antisense lncRNA GABPB1-AS1 in erastin-induced ferroptosis, and established it as an attractive target for HCC treatment^24^. In brief, lncRNAs tend to be promising novel diagnostic and prognostic markers for HCC.

It is vital to find the beneficial people for immunotherapy and evaluate the efficacy of immunotherapy so that the immune checkpoint inhibitors can be chosen accurately in the population. In our study, we established a novel signature of ferroptosis-related lncRNAs, which was developed to predict the OS of HCC patients. We then explored the immune infiltration based on the prognostic signature. In short, the novel signature may serve as a reference for future investigations at the dawn of a promising new era of HCC treatment.

## Materials and methods

### Preparation of information of patients with HCC, selection of ferroptosis-related lncRNA

We obtained RNA-sequence (normal and tumor) transcriptome data and clinic-pathological data of LIHC patients from The Cancer Genome Atlas (TCGA) (https://www.cancer.gov/) database. To avoid statistical bias in this study, we excluded patients with incomplete clinical information. We downloaded ferroptosis-related genes from FerrDb (http://www.zhounan.org/ferrdb/), including 108 drivers, 69 suppressors, and 111 markers^25^. We performed a Pearson correlation analysis to assess the relationship between the ferroptosis-related genes and lncRNAs. The association has completed the criteria |R^2^|> 0.3 and *p* < 0.001.

### Establishing a prognostic signature to evaluate the riskScore

The entire TCGA set was randomly divided into training and testing sets. The training set was performed to establish the ferroptosis-related lncRNAs signature, and the entire TCGA set and testing set were performed to verify the reliability of the signature. There was no significant statistical difference in clinical characteristics between the training and testing sets. We first performed a univariate Cox regression analysis (*p* < 0.01) to examine the prognosis of ferroptosis-related lncRNAs. Then, we conducted Least absolute selection operator (LASSO) Cox regression by using R package *glmnet* (performing the penalty parameter calculated by tenfold cross-validation and a *p*-value of 0.05). We found that 60 ferroptosis-related lncRNAs were associated with OS in HCC patients in the entire TCGA set. Then, 11 ferroptosis-related lncRNAs were obtained after LASSO Cox regression analysis of 60 ferroptosis-related lncRNAs. Among them, 11 ferroptosis-related lncRNAs were included in the multivariate Cox regression analysis, and a 6 ferroptosis-related prognostic signature was established. We calculate the riskScore according to the following formula. RiskScore = (coef lncRNA1 × expr lncRNA1) + (coef lncRNA2 × expr lncRNA2) + ⋯ + (coef lncRNAn × expr lncRNAn). Coef lncRNAn represents the correlation between lncRNA and survival of HCC patients. Expr lncRNAn represents the expression level of lncRNA. The RNAs were classified in either low-risk (< median number) or high-risk (≥ median number) groups based on the median score.

### Validation of the ferroptosis-related lncRNAs prognostic signature

To validate the riskScore, we conducted Kaplan–Meier survival analysis to reveal the survival difference of HCC patients in the high- or low-risk group, and the survival curve was performed for visualization. We used Rtools to visualize the specific riskScore for each signature sample. The relationship between ferroptosis-related lncRNAs and clinic-pathological characteristics was evaluated using logistic regression and a heatmap graph. The sensitivity and specificity of the prognostic signatures for HCC patients compared to other clinic-pathological characteristics were assessed utilizing the receiver operating characteristic curve (ROC) and decision curve analysis (DCA). R packages utilized in these steps included *survival*, *surviminer*, *pHeatmap*, *timeROC*, *ggDCA*.

### A network diagram of lncRNA-mRNA, and independence of the prognostic signature

A total of 6 ferroptosis-related lncRNAs which constructed the novel signature formed a network diagram interrelated with ferroptosis-related mRNAs. Univariate (*p* < 0.05) and multivariate (*p* < 0.05) Cox regression analyses were utilized to confirm whether the prognostic signature can be used as an independent clinical prognostic predictor considering other clinicopathological characteristics (age, gender, grade, TNM stage) in the patients with HCC. Forest maps were constructed to visualize the results. The procedure was performed using R *survival* packages and Cytoscape 3.7.0.

### Establishing a predictive nomogram

A nomogram was constructed integrating the prognostic signatures (age, gender, grade, TNM stage, T stage, N stage, M stage) for the 1-, 3-, and 5‐year OS of patients with HCC. The procedure was performed using R packages, including *survival*, *regplot*.

### Exploration of gene set enrichment

We performed Gene set enrichment analyses (GSEA) to define the lncRNAs signatures in the KEGG^26^, which were then searched in the TCGA-HCC database. Statistical significance was set at *P* < 0.05 and false discovery rate (FDR) q < 0.05. The procedure was performed using R packages included *plyr*, *ggplot2*, *grid*, *gridExtra*.

### Analysis of tumor-infiltrating immune cells

Tumor-infiltrating immune cell dataset was downloaded in TIMER2.0 (http://timer.cistrome.org) to download. The TIMER, CIBERSORT, quanTIseq, MCP-counter, xCELL, and EPIC algorithms were simultaneously compared to evaluate cellular components or cellular immune responses between high-risk and low-risk groups based on ferroptosis-related lncRNAs signature. The differences in the immune response under different algorithms were uncovered using a heatmap. Moreover, ssGSEA was conducted to quantify the subgroup of tumor-infiltrating immune cells between the high- and low-risk group and estimate their immune function. Potential immune checkpoints were collected from previous literature and evaluated the differences in expression between the two groups. R packages utilized in these steps included *limma*, *ggpubr*, *pHeatmap*, *reshape2*, *GSEABase*, *ggplot2*.

## Results

### Identification of ferroptosis-related lncRNAs in patients with LIHC

**Figure 1:**
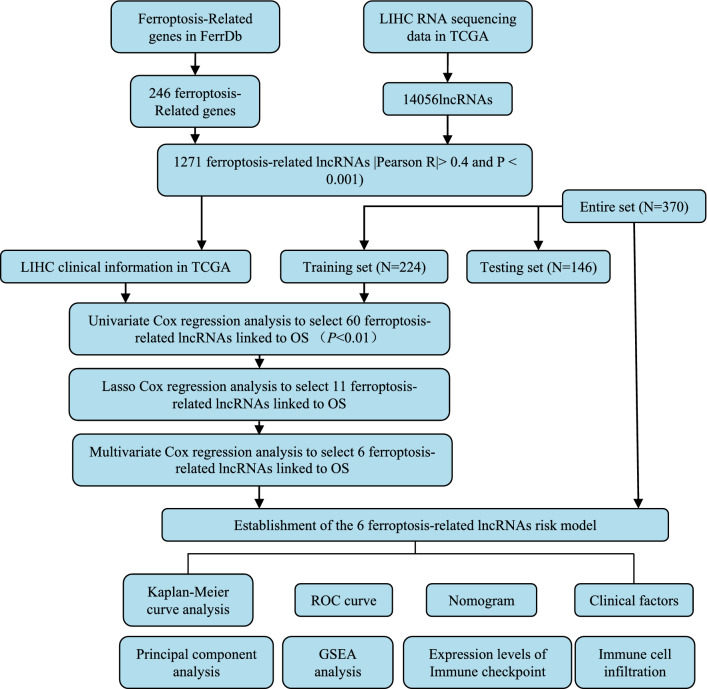
Workflow of this study.

The workflow of prognostic signature construction in this study is shown in Fig. 1. First, we abstract 14,056 lncRNAs and 259 ferroptosis-related genes (driver: 108, suppressor: 69, marker: 111) (Table S1) from the liver hepatocellular carcinoma (LIHC) project of the TCGA database. Next, 1,271 ferroptosis-related lncRNA was obtained by performing Pearson correlation analysis between lncRNAs and ferroptosis-related genes (Table S2).

### Construction and validation of a prognostic signature according to ferroptosis-related lncRNAs in LIHC patients

**Figure 2:**
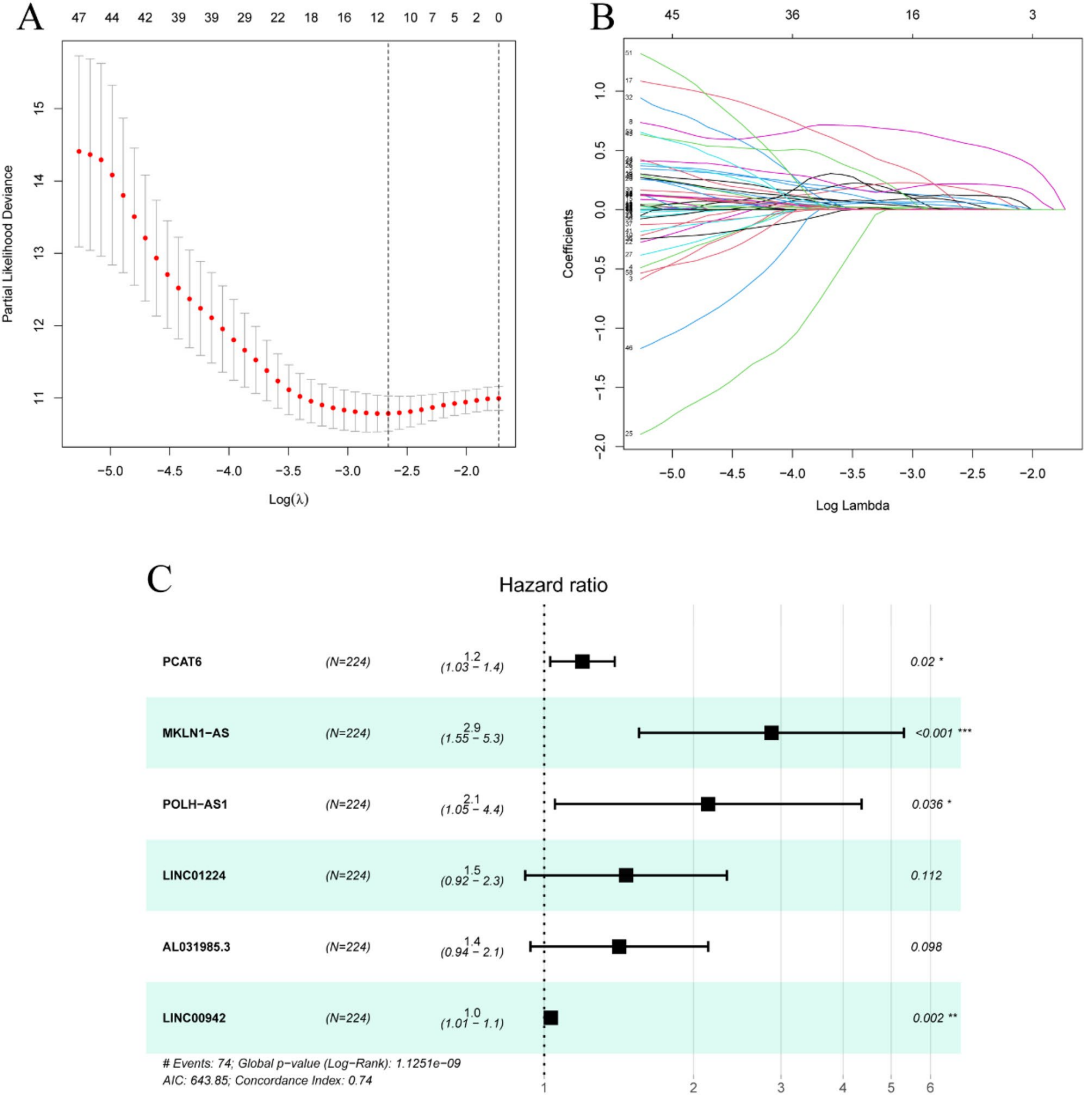
Six ferroptosis-related lncRNAs were selected to establish a prognostic signature. (**A**) Selection of the optimal turning parameters (log λ) through the tenfold cross-validation. (**B**)The Lasso coefficient profile of 60 OS-related lncRNAs and imaginary perpendicular lines were drawn at the value chosen by tenfold cross-validation. (**C**) Multivariate Cox regression analysis showed 6 ferroptosis-related lncRNAs.

Ferroptosis-related prognostic lncRNAs from 1271 ferroptosis-related lncRNAs were screened using univariate Cox regression analysis in the TCGA training set. A total of 60 ferroptosis-related lncRNAs in the training set were correlated with OS (Table S3). LASSO Cox regression analysis is a compression estimation method based on reducing variable sets. This method can compress the variable coefficient and make some regression coefficients turn to 0 by constructing a penalty function to achieve the purpose of variable selection. 11 ferroptosis-related lncRNAs were obtained by Lasso Cox regression analysis for multivariate Cox regression analysis (Fig. 2A–C). Subsequently, we performed multivariate Cox regression analysis to identify prognostic ferroptosis-lncRNAs. 6 ferroptosis-lncRNAs (PCAT6, MKLN1-AS, POLH-AS1, LINC00942, AL031985.3, LINC00942) in the training set were considered prognostic proteins associated with OS (Table S4). They were performed to establish a predictive signature for LIHC patients in the training set.

### Survival results and multivariate examination

**Figure 3:**
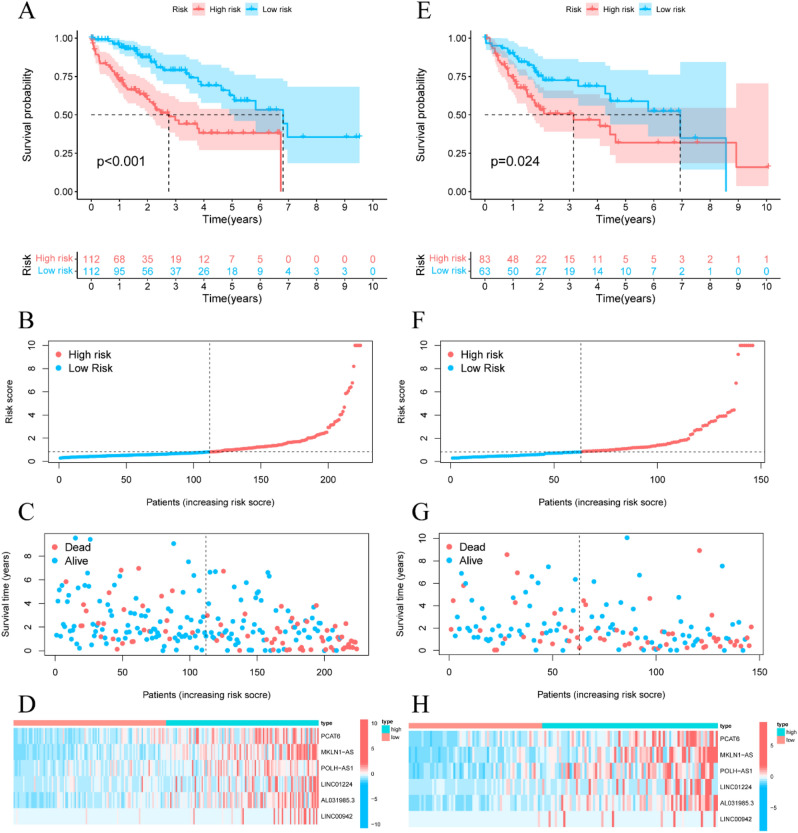
Prognostic signature of the riskScore analyses of the 6 ferroptosis-related lncRNAs in the TCGA training and testing sets. (**A**) Kaplan–Meier survival curves of the OS of patients were ranked by riskScore for the training set. (**B**) Distribution of ferroptosis-related lncRNA model-based riskScore for the training set. (**C**) Patterns of the survival time and survival status were ranked by riskScore. (**D**) Clustering analysis heatmap shows the display levels of the 6 lncRNA for each patient in the training set. (**E**) RiskScores ranked Kaplan–Meier survival curves of the OS of for the testing set. (**F**) Distribution of ferroptosis-related lncRNA model-based riskScore for the testing set. (**G**) Patterns of the survival time and survival status were ranked by riskScore. (**H**) Clustering analysis heatmap shows the display levels of the 6 lncRNA for each patient in the testing set.

HCC patients in the training set were divided into high- and low-risk groups based on the median value of the predictive riskScore. Figure 3A revealed that the survival time of HCC patients was significantly longer in the low-risk group than in the high-risk group (*P* < 0.001). The distribution of prognostic signature is shown in Fig. 3B, and the survival outcomes of patients in different groups are shown in Fig. 3C. The expression profiles of the 6 ferroptosis-related lncRNAs are shown in Fig. 3D.

**Figure 4:**
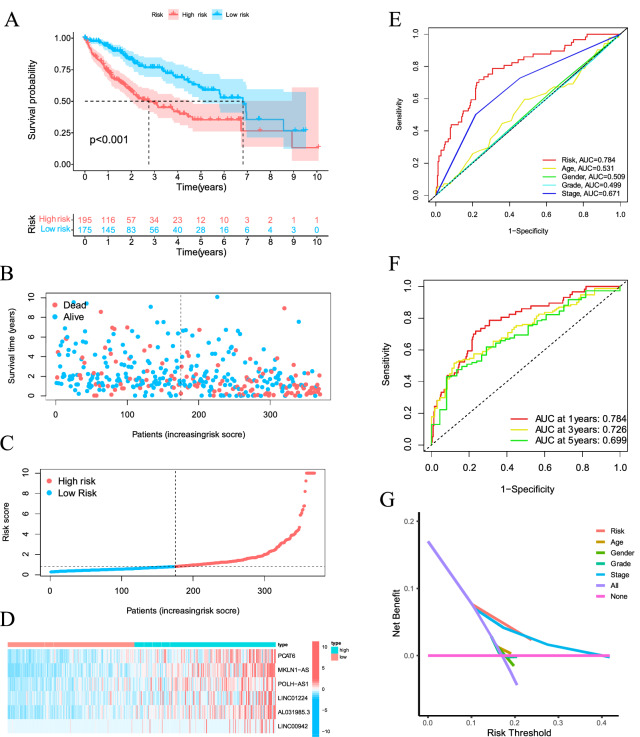
Ferroptosis-related lncRNAs prognostic signature based on TCGA entire set. (**A**) Kaplan–Meier survival curves of the OS of patients were ranked by riskScore for the entire set. (**B**) Distribution of ferroptosis-related lncRNA signature was based riskScore for the entire set. (**C**) Patterns of the survival time and survival status between the high- and low-risk groups for the training set. (**D**) Clustering analysis heatmap shows the display levels of the 6 lncRNA for each patient in the training set. (**E**) The AUC values of the risk factors. (**F**) The AUC for the prediction of 1, 3, 5-year survival rate of LIHC. (**G**) The DCA of the risk factors.

To verify the accuracy of this established signature, we analyzed riskScore in the validation set and entire set using uniform formula. Kaplan–Meier survival analysis, risk distribution, survival outcomes, and expression of the ferroptosis-related lncRNAs were shown in the validation set (Fig. 3E–H) and entire set (Fig. 4A–D).

### Evaluation of the prognostic signature of ferroptosis-related lncRNAs and clinical features of HCC

The area under curve (AUC) of the risk grade was higher than the AUCs of other clinicopathological features, indicating that the prognostic signature of the 6 ferroptosis-related lncRNAs for LIHC was reasonably dependable (Fig. 4E). The AUC of the novel lncRNAs signature for 1-, 3-, 5-year survival rates was 0.784, 0.726, 0.699, respectively (Fig. 4F). Decision Curve Analysis (DCA) showed that the line of prognostic signature and stage stay away from the lines of other clinicopathological features, indicating both prognostic signature and TNM stage can remarkably predict the prognosis of LIHC (Fig. 4G).

**Figure 5:**
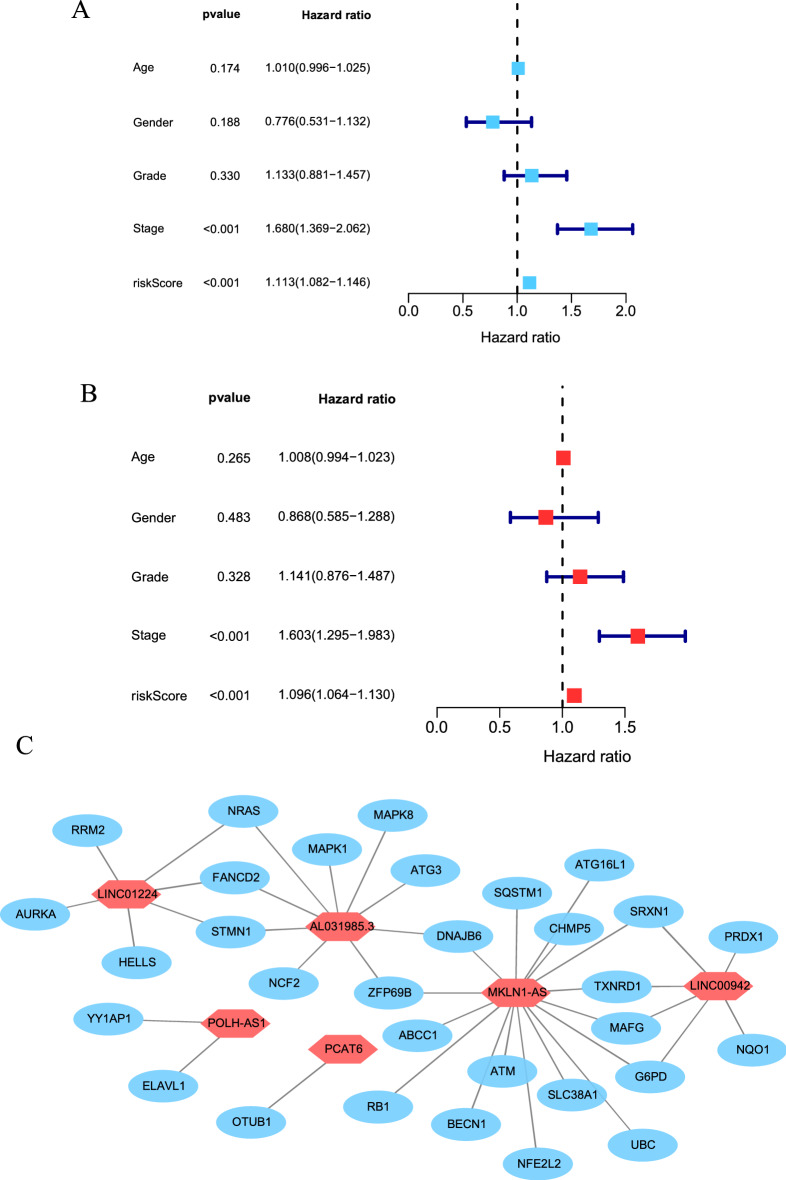
Assessment of the prognostic signature of the ferroptosis-related lncRNAs. (**A**) Univariate Cox regression analysis of the clinical characteristics and riskScore with the OS. (**B**) Multivariate analysis of the clinical characteristics and riskScore with the OS. (**C**) The relationship between the novel lncRNA and mRNA expression.

Univariate and multivariate Cox regression analyses revealed that the prognostic signature of 6 ferroptosis-related lncRNAs were independent prognosis factors for LIHC. Univariate Cox regression showed that the HR was 1.096 and 95% CI was 1.064–1.130 (*P* < 0.001), respectively (Fig. 5A). Multivariate Cox regression showed that the HR was 1.113 and 95% CI was 1.082–1.146 (*P* < 0.001), respectively (Fig. 5B). The relationship between ferroptosis-related lncRNA and ferroptosis-related mRNA is shown in Fig. 5C.

### Principal-component analysis (PCA) further verifies the grouping ability of the ferroptosis-related lncRNA signature

PCA was performed to prove the difference between high-risk and low-risk groups based on the entire gene expression profiles, 246 DE ferroptosis-related genes, 6 ferroptosis-related lncRNAs, and prognostic signature classified by the expression profiles of the 6 ferroptosis-related lncRNAs.

**Figure 6:**
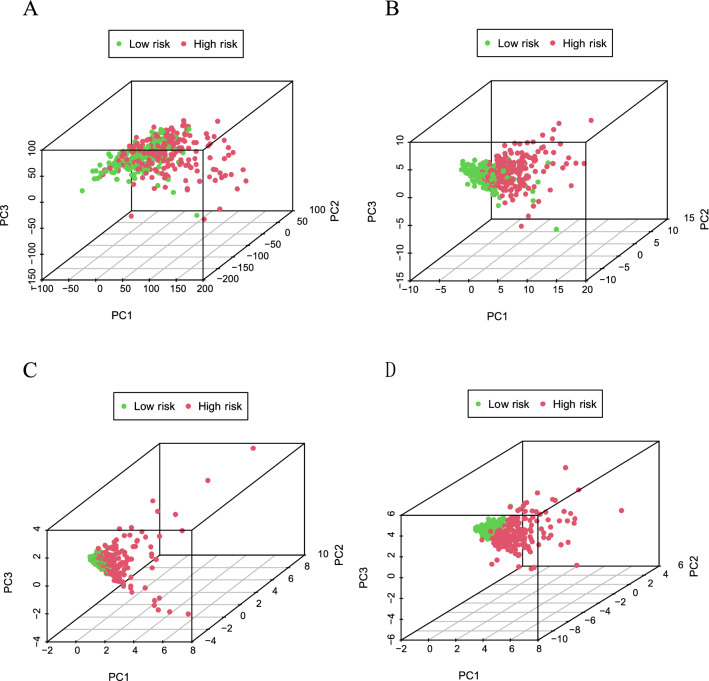
Principal component analysis between the high and low-risk groups in TCGA entire set. (**A**) entire gene expression profiles, (**B**) ferroptosis-related genes, (**C**) ferroptosis-related lncRNAs, (**D**) prognostic signature based on ferroptosis-related lncRNAs.

Figure 6A–C shows that the distribution of high-risk and low-risk groups was relatively dispersed. However, the results derived from our signature indicate that high-risk and low-risk groups have different distributions (Fig. 6D). These results manifest that the prognostic characteristics can distinguish between high-risk and low-risk groups.

### Nomogram and heatmap of clinical factors

**Figure 7:**
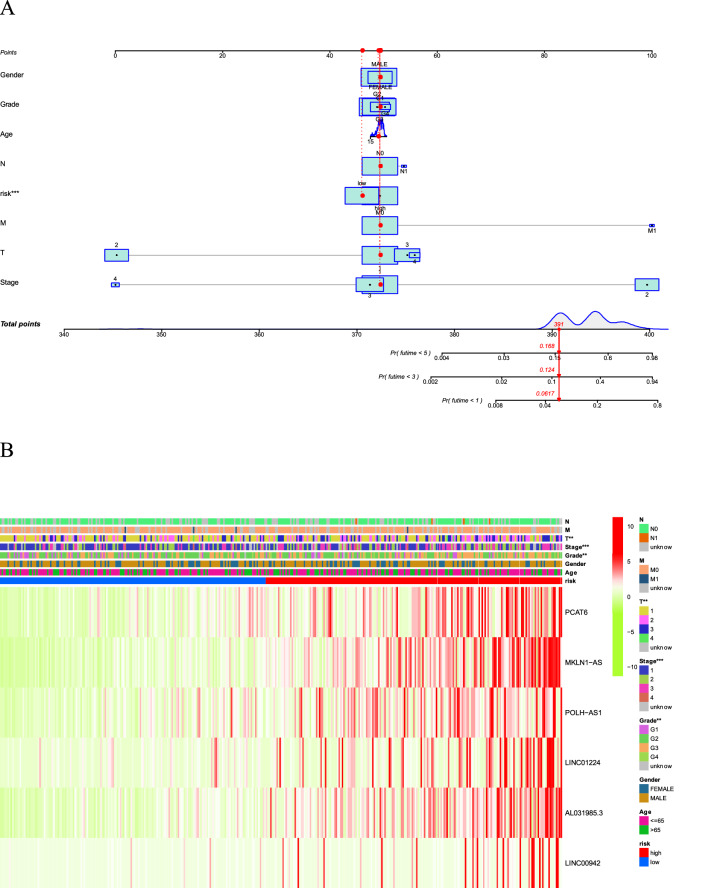
(**A**) A nomogram for both clinic-pathological factors and prognostic ferroptosis-related lncRNAs. (**B**) Heatmap for ferroptosis-related lncRNAs prognostic signature and clinic-pathological characteristics.

Figure 7A shows a stable and accurate hybrid nomogram that contains clinic-pathological characteristics and the novel prognostic signature. Thus, the prognostic signature may be applied in the clinical management of HCC patients. The heatmap for the association between ferroptosis-related lncRNAs prognostic signature and clinic-pathological characteristics were also analyzed (Fig. 7B).

### Gene set enrichment analyses

**Figure 8:**
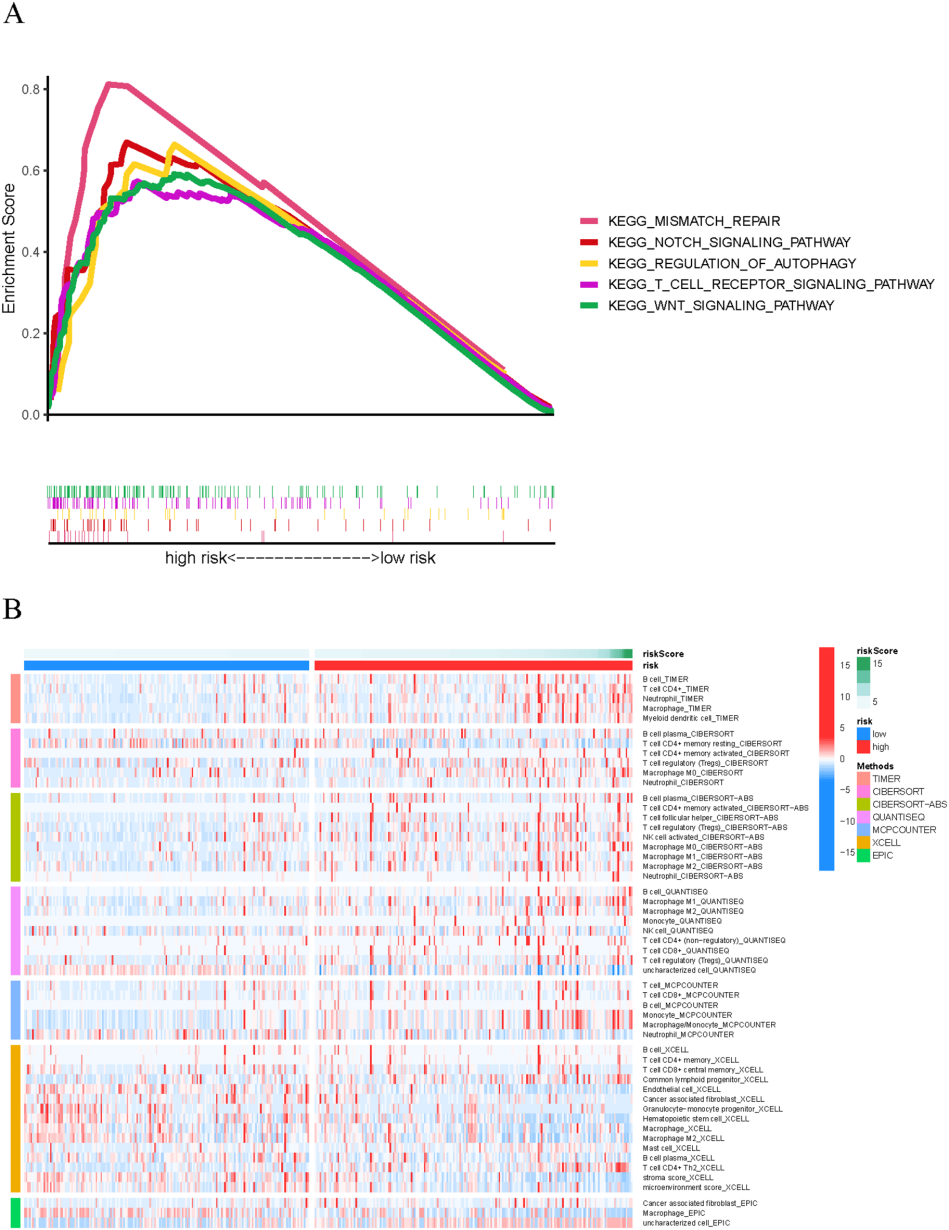
(**A**) Gene enrichment analysis for ferroptosis-related lncRNAs based on TCGA entire set. (**B**) Heatmap for immune infiltration based on TIMER, CIBERSORT, quanTIseq, MCP-counter, xCELL, and EPIC algorithms among high- and low-risk groups.

GSEA revealed that most novel ferroptosis-related lncRNAs prognostic signature regulated immune and tumor-related pathways such as T cell receptor signaling pathway, mismatch repair, Notch signaling pathway, regulation of autophagy, Wnt signaling pathway (Fig. 8A) and (Table S5).

### Estimation of the tumor immune microenvironment and immune checkpoints using the ferroptosis-related lncRNA signature

**Figure 9:**
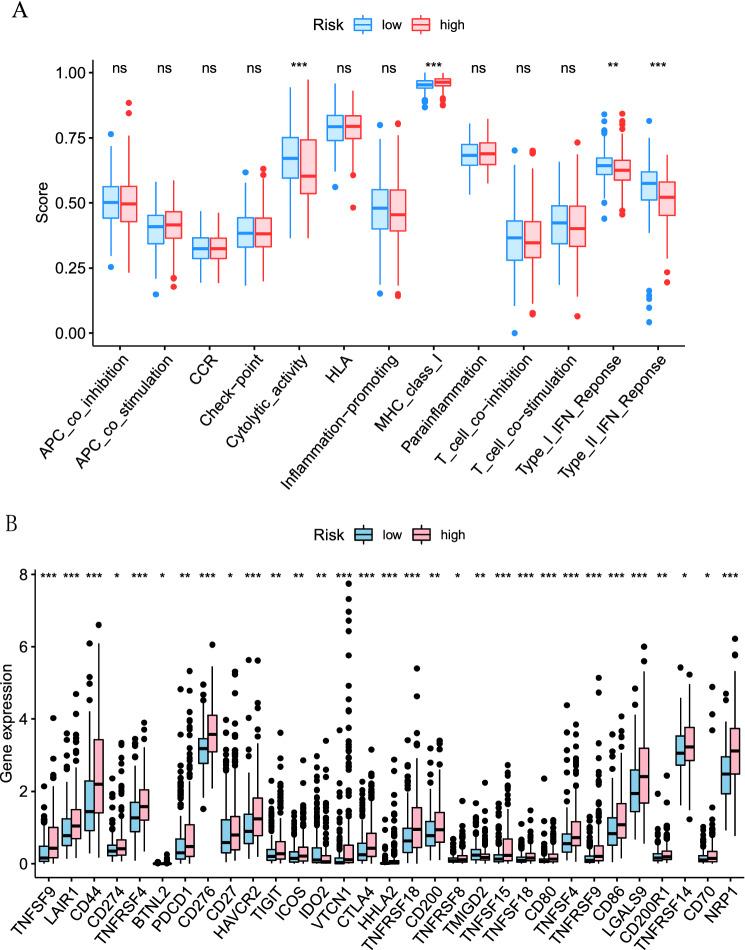
(**A**) ssGSEA for the association between immune cell subpopulations and related functions (**B**) Expression of immune checkpoints between high- and low- risk groups.

The heatmap of immune responses based on TIMER, CIBERSORT, quanTIseq, MCP-counter, xCELL, and EPIC algorithms is shown in Fig. 8B. Correlation analysis between immune cell subpopulations and related functions based on ssGSEA of TCGA-LIHC data revealed that immune cell subpopulations and related functions including cytolytic activity, MHC class I, type I, and type II IFN response were significantly different between high-risk and low-risk groups Fig. 9A. Given the importance of immune checkpoints in immunotherapy, we further explored the differences in immune checkpoint expression between the two groups. We found a substantial difference in the expression of PDCD1, CTLA4, CD44, VTCN1, among others, between two groups of HCC patients (Fig. 9B).

## Discussion

The high incidence and mortality of HCC have attracted many researchers to lucubrate its development and treatment. Existing studies have shown that disparate subtypes of HCC have different clinical features and prognoses. Nowadays, increasing studies have focused on non-coding RNAs to explore their influence on prognosis and immune response in patients with HCC^27–29^. Similarly, the resistance of tumor cells to anti-tumor drugs has led researchers to focus on programmed cell death, such as autophagy, pyroptosis, ferroptosis. Ferroptosis is a new type of cell death that can potentially provide a valuable approach to cancer treatment. As we know, sorafenib has been identified as an inducer of ferroptosis and has long occupied the first-line treatment of HCC. However, the status of sorafenib has been shaken by the emergence of immunotherapy as a first-line treatment. Our study investigated the effects of ferroptosis and lncRNAs on the immunity of HCC. In this study, we determined the prognostic characteristics of ferroptosis-related lncRNAs in the LIHC dataset of TCGA. Then, we validated differences in immune-infiltrating cells and immune checkpoints in the tumor microenvironment based on the novel prognostic signature.

In our study, 781 ferroptosis-related lncRNAs were identified from the TCGA dataset to investigate the prognostic indicator of ferroptosis-related lncRNAs. The TCGA dataset confirmed the predictive value of 11 ferroptosis-related lncRNAs, 6 of which were performed to construct signatures to predict OS in HCC patients. 4 of the 6 lncRNAs, PCAT6, MKLN1-AS, POLH-AS1, LINC00942, were independent prognostic factors associated with survival in HCC patients. Studies showed that PCAT6 regulates cell proliferation and migration of HCC by blocking the cell cycle^30^. Gao et al.^31^ discovered MLKN1-AS acted as a molecular sponge for miR-654-3p to increase HDGF expression and further induce the progression of HCC. POLH-AS1 is one of the super-lncRNA that target super-enhancers, and its function remains unknown. Sun et al.^32^ found that LINC00942 promoted the proliferation and progression of breast cancer cells by upregulating m6A methylation mediated by METL14. However, the function of LINC00942 on HCC remains unknown. Therefore, the functions of POLH-AS1 and LINC00942 in HCC remain to be clarified. We performed the selected 6 lncRNAs to establish a novel prognostic signature. HCC patients were divided into high-risk group and low-risk group according to the median score. Clinical outcomes in the high-risk group were significantly worse. In multivariate Cox regression analysis, the prognostic signature established by ferroptosis-related lncRNA was an independent risk factor for HCC prognosis. ROC analysis showed that the signature outperformed conventional clinical features in predicting survival in HCC patients. In addition, we established a nomogram showing ideal consistency in 1-, 3-, and 5-year prediction rates. The prognostic signature is reliable and accurate and can identify new biomarkers for subsequent studies.

Compared with mRNA, lncRNA possesses higher specificity of tissue and organs. As an important signal molecule in the human body, lncRNAs are involved in transcriptional silencing, transcriptional activation, chromosome modification, nuclear transport, and other processes^33^. Ferroptosis-related studies have attracted attention from numerous cancer fields. It is well known that lncRNAs participate in the activity of cancer cells. However, in recent years, it has been proved that lncRNAs are involved in regulating the ferroptosis of cells, and some novel ferroptosis-related mechanisms have been discovered. Mao demonstrated that cytosolic lncRNA P53RRA functions as a tumor suppressor by activating the p53 pathway and promoting ferroptosis and apoptosis in cancer^23^. Qi and colleagues^24^ found that Hep G2 HCC cells treated with Erastin can promote ferroptosis of HCC cells through the GABPB1-AS1/GABPB1/PRDX5 axis. However, studies on the role of ferroptosis and lncRNAs in HCC progression remain limited. Studies on predictive biomarkers and biological mechanisms of ferroptosis-related lncRNAs in HCC are still scarce. In this study, we were inspired by the role of ferroptosis and lncRNAs in HCC; thus, we attempted to establish a novel signature based on ferroptosis-related lncRNAs. In this study, we substituted the ferroptosis-related lncRNA related to the prognosis into the formula for risk scoring and divided patients into high-risk and low-risk categories to explore the potential role of the novel signature in HCC.

Immunotherapy, the most promising anti-tumor treatment, strengthens the therapeutic effect by activating the immune function. However, autoimmune toxicity and immune escape caused by immunotherapy are the maximal obstacles to tumor immunotherapy^34^. Therefore, it is crucial to enhance immune cells recognizing tumor cells. Recently, accumulating studies have proved that ferroptosis is related to immune regulation, such as, CD8(+) T cells can increase the anti-tumor effect by inducing ferroptosis^35,36^. CD8(+) T cells promote lipid peroxidation and ferroptosis by inhibiting the expression of SLC7A11 and SLC3A2 transporters by releasing interferon γ and reducing cystine uptake by tumor cells^7^. Studies have found that iron ions regulate the expression of cyclin E1 and promote the proliferation of B cells by regulating demethylases^37^. In addition, compared with M2 macrophages, M1 macrophages are affected by inducible nitric oxide synthase and exhibit antagonism to ferroptosis^38^. They proved that the role of the nitric oxide pathway in inhibiting the ferroptosis of macrophages contributes to enhancing the anti-tumor immunity of M1 macrophages.

We routinely consider pathological staging as a prognostic factor of HCC in clinical practice. However, HCC patients with the same pathological stage may have different clinical outcomes. This phenomenon shows that according to the pathological staging, assessing and predicting the heterogeneity of HCC is not reliable Therefore, many scholars are committed to discovering biomarkers with potential predictive and therapeutic value. The ferroptosis-related lncRNA signature established in our research provides new ideas for evaluating the prognosis of HCC patients.

In conclusion, our study provides references for prognosis prediction of HCC patients and is conducive to clarifying the process and mechanism of lncRNA in ferroptosis of HCC in the future. Although the results of our study were grouped and validated, the prognostic signature developed in this study still needs to be further validated.

## Supplementary Information

Supplementary Information.

## Data Availability

All raw and processed data are freely available from TCGA database (https://www.cancer.gov/).

## Abbreviations

TCGA: The cancer genome atlas
HCC: Hepatocellular carcinoma
RFA: Radiofrequency ablation
OS: Overall survival
PFS: Progression free survival Long-noncoding RNAs lncRNAs
ORF: Open reading frame
TACE: Transcatheter arterial chemoembolization
LIHC: Liver hepatocellular carcinoma
AUC: Area under curve
DCA: Decision curve analysis
GSEA: Gene set enrichment analyses
ROC: Receiver operating characteristic
LASSO: Least absolute selection operator
PCA: Principal component analysis
FDR: False discovery rate
